# Towards a More Nuanced View of Vocal Attractiveness

**DOI:** 10.1371/journal.pone.0088616

**Published:** 2014-02-19

**Authors:** Molly Babel, Grant McGuire, Joseph King

**Affiliations:** 1 Department of Linguistics, University of British Columbia, Vancouver, British Columbia, Canada; 2 Department of Linguistics, University of California, Santa Cruz, California, United States of America; University of Sussex, United Kingdom

## Abstract

This study reports on male and female Californians' ratings of vocal attractiveness for 30 male and 30 female voices reading isolated words. While ratings by both sexes were highly correlated, males generally rated fellow males as less attractive than females did, but both females and males had similar ratings of female voices. Detailed acoustic analyses of multiple parameters followed by principal component analyses on vowel and voice quality measures were conducted. Relevant principal components, along with additional independent acoustic measures, were entered into regression models to assess which acoustic properties predict attractiveness ratings. These models suggest that a constellation of acoustic features which indicate apparent talker size and conformity to community speech norms contribute to perceived vocal attractiveness. These results suggest that judgments of vocal attractiveness are more complex than previously described.

## Introduction

The voice is a rich source of information for listeners. In addition to functioning as the medium of communication in oral language, in its non-linguistic role, the human voice has the ability to convey biological information like sex (e.g., [1]) and age (e.g. [2]); physiological details such as height and weight for men [3]; social classifications such as race [4]; and emotional states [5]. The attractiveness of a particular voice is potentially related to a number of these talker-specific physical and social assessments. Previous work on vocal attractiveness has used a small selection of acoustic-phonetic measures that are related to vocal tract size to predict listeners' judgments of attractive voices. In this study, we employ a larger range of phonetic measures related to both the apparent size of talkers' laryngeal source and supralaryngeal cavity, and non-physiological stylistic aspects of spoken language measurable from the signal to study the subjective vocal attractiveness ratings of sixty talkers.

Attractiveness, as a general topic, is of interest for many reasons, and one major perspective is that human physical attraction drives the selection of mate partners [6]. For vocal, rather than physical, attractiveness, this theory is supported by a body of research linking vocal traits to human sexuality and dimorphism. For example, there is evidence that women's preferences for masculine-sounding men are enhanced during the fertile menstrual cycle phase [7] [8], that voice preference and bilateral symmetry are linked [9] [10], and that facial and vocal attractiveness are linked [11] [12] [13] [14]. Moreover, traits that are usually linked to reproductive success in other mammals and non-human primates have been found to be connected with vocal traits, such as dominance [15] and threat potential [14]. Some researchers have even underscored the importance of understanding vocal attractiveness because of its importance to sexual selection in nocturnal copulation [16] (but see [17]). Vocal attractiveness is an important social evaluation that goes beyond mate selection and sexual behavior. Judgments of attractiveness are important in everyday interaction as physically attractive people are judged to be more socially desirable and to get better jobs [18], in addition to being more persuasive [19].

Given that much of the vocal attractiveness literature emphasizes the role of mate selection and sexual preferences, there is justifiably a strong focus on voice features relating to sexual dimorphism in humans—especially fundamental frequency and measures related to vocal tract length, such as formant dispersion. The former of these two features has been well established in its relationship with vocal attractiveness. Fundamental frequency (f0) is an acoustic measure of the rate of vibration of the vocal folds and the primary acoustic property that listeners perceive as vocal pitch. Longer vocal folds tend to vibrate at a lower rate, typically giving males their lower pitch, whereas the vocal folds of females, being shorter, naturally vibrate at a higher rate than males', providing a generally higher-pitched voice. Within English the general consensus is that a slightly higher-than-average overall f0 is considered more attractive for female voices and that a slightly lower-than-average voice is more attractive in male talkers [12] [13] [20] [21] [22] [23] [24]. This preference for lower-pitched male voices holds for both adolescent and adult-aged females, although not female children [12]. This finding is hypothesized to be a reinforcing or an exaggeration of the average laryngeal differences between males and females and is thought to have cross-cultural relevance despite the degree of apparent size and actual size difference between males and females varying across cultures ([25] [26] [27]; see [28] for a discussion of the role of male and female height differences across languages as related to male and female differences in formant frequencies). For example, van Bezooijen [29] proposes that the reason pitch differences between males and females are greater in Japanese than in Dutch is due to greater relative extremes in gender stereotypes and expectations, but that basic interpretations of higher and lower f0 are culturally universal. In a study of Hadza speakers living as hunter-gatherers in Tanzania, lower-pitched males had higher levels of reproductive success, suggesting the existence of selectional pressures for low-pitched male voices in such communities [30].

These various effects of f0 were extensively tested in a large-scale study of female voices, Feinberg et al. [24], which confirmed that higher pitched voices were rated as more attractive than lower-pitched female voices. A subset (n = 15) of the female voices – five each from a low pitch group (200 Hz), an average group (220 Hz), and a high group (240 Hz) – were selected for Feinberg and colleagues' second study which involved manipulating f0 so as to modify apparent larynx size. Using a forced-choice paradigm, male listeners judged attractiveness, age, and femininity from the vocal samples. Listeners judged talkers with apparent smaller larynxes to sound more feminine and younger. Voices with raised f0 were rated as more attractive by male listeners than those without raised f0; this effect was strongest for the voices in the low pitch group.

There is less consensus regarding the role of overall vocal tract size in attractiveness ratings. For example, Hodges-Simeon et al. [31] found that males with less dispersed formants (suggesting a longer vocal tract) were preferred by fertile-phase women (as well as low fundamental frequencies being more generally preferred). Other studies have directly explored these effects through modifications of the formant frequencies of natural voices to change f0 and vocal tract size, both independently and simultaneously. With male voices, Feinberg et al. [8] found that voices with lowered fundamental frequencies were rated as more attractive, but no effect was found from manipulating formant frequencies.

Pisanski and Rendall [32] offer another comparison of f0 and resonance characteristics. Their study also manipulated both values using a metric to establish JNDs for listener populations for each vocal feature. When compared, resonance characteristics were found to be weighted more heavily than f0 in determining attractiveness, size, and masculinity for males; the results for females were more mixed.

Some suggest that attractiveness ratings may relate to other qualities deducible from the voice, such as dominance [15]. Puts, Gaulin, and Verdolini [23] examined the perception of social and physical dominance in male voices by independently manipulating f0 and the dispersion of formant frequencies. Both larger apparent vocal tract length and larger apparent larynx size resulted in higher dominance ratings, but the effect of apparent vocal tract length affected judgments of physical dominance more than social dominance. Later work from the same group has found similar results with a more reliable measure, standardized formant position [14]. This measure was more strongly associated with sexual dimorphism and height than formant spacing in samples of participants from the United States and a Hadza community.

However, it is important not to overstate the relationship between formant measures and body size as these measures are only weakly related, and listeners are not adept at making fine judgments in speaker size [33]. González [34], for example, provides data from Spanish speakers which illustrate that the relationship between formant frequencies and body size is extremely tenuous. While it is possible that non-linguistic vocalizations may be more indicative of talker size, the primacy of linguistic communication in humans argues for acoustic-phonetic information that is more robustly carried through that medium to be the primary source of dominance and attractiveness judgments. Measures such as f0 and formant spacing seem to derive more from masculine and feminine traits extrapolated from the physiology.

One additional phonetic characteristic has been shown to be relevant to judgments of vocal attractiveness: voice onset time (VOT). VOT is a temporal descriptor of oral stops when they are followed by a voiced sound (e.g., a vowel) that measures the duration, either positive or negative, of the lag interval between the onset of vocal fold vibration and the release of the oral closure. VOT has been demonstrated to vary during women's menstrual cycle such that those who are at their reproductive peaks have longer VOTs than those at their lowest fertility levels [35] [36], which would increase the clarity of the contrast between, for example, a /b/-initial word like *bad* and a /p/-initial word like *pad*. This is related to the similar observation that women at reproductive peaks of their cycle are rated as more vocally attractive [16] [37]. These results suggest that, perhaps, measures of speech clarity influence attractiveness judgments as well.

Beyond individual acoustic properties of voices, Bruckert et al. [38] examined the role of averageness in attractiveness. This is a well-established phenomenon in visual attractiveness where the merging of various faces into a single composite face results in a more attractive face than most (or all) of the component ones (e.g. [39]). With respect to speech, Bruckert et al. [38] devised an innovative method of merging voices in an analogous way and found a similar result—i.e. the more voices merged, the higher the overall attractiveness rating. Further exploration of this result demonstrated that averaging the voices resulted in “smoother” voices. Their measure for smoothness was harmonic-to-noise (HNR) ratio; the higher the HNR the less hoarse a voice sounds [40]. Thus, voices with higher HNRs were more attractive. Moreover, and somewhat in conflict with previous results, more typical f0 and F1 values, with respect to each gender, were more attractive. The reason for the discrepancy between these results and those of Feinberg et al. [8], for example, is not clear.

To summarize, there is evidence that acoustic measures derived from sexual dimorphism, such as f0, play a significant role in judgments of vocal attractiveness. The voice spectrum is very complex and many other phonetic characteristics not previously included in studies of vocal attractiveness could contribute significantly to such judgments. Presumably any acoustic feature that may signal sex or social differences may be a significant predictor of vocal attractiveness. Previous research seems to underplay the performative aspects of spoken communication – speech is learned and used in a way that reflects identity construction, part of which might involve the use of more prescriptive gender norms, which echoes sexual dimorphic traits. In the experiment described below, we present evidence that various acoustic qualities which are potentially related to a talker's apparent size, apparent health and youthfulness, and membership in a community contribute to judgments of vocal attractiveness. To do this we used recordings of monosyllabic words, which we see as an improvement over studies that examined single vowels (e.g., [8]), as real word production is more ecologically valid for both the talkers and the listeners. In terms of pinpointing aspects of the acoustic signal that cue judgments of vocal attractiveness, single words are more appealing than full sentences, as they allow for a more controlled acoustic analysis.

In addition to the previously examined voice features such as f0 and formant spacing, this study integrates several more features that are known to systematically vary by gender, namely voice quality and duration. Voice quality is largely determined by glottal source characteristics [41] and the thinner, less massive vocal folds of women result in overall breathier voices [42] [43] [44]. This aspect of sex specific difference has been largely ignored in the vocal attractiveness literature with a few notable exceptions. The aforementioned Bruckert et al. study examined HNR, which is known to correlate with voice quality, especially hoarseness [40]. The results of that study imply that more regular vocal fold vibration was more attractive and this pattern held for both male and female voices. As to the dimorphic properties of voice quality, breathiness has been argued to be a feminine trait and related to desirability in women [45]. Based on these facts we predict that breathier female voices (within norms) would be judged to be more attractive. However, one previous attempt at examining voice quality using measures such as jitter and shimmer (consistency of rate and amplitude of vocal fold vibration, respectively) did not show clear results [10]. Other measures of voice quality remain untested.

Additionally, speech clarity, which was mentioned above with respect to VOT, is generally described as a female trait which may be used to assess attractiveness. Bradlow, Torretta, and Pisoni [46] demonstrate that female talkers produce sentences with a more expanded vowel space, less reduction, and longer durations. These factors are less directly related to physiology than f0 and voice quality, but may have some relation to speech dynamics resulting from smaller female vocal tracts [47] [48]. To our knowledge the only study to examine duration effects is Hughes et al. [10], and they were unsuccessful.

The goal of the present study, then, is to explore a wider variety of acoustic measures as predictors of attractiveness, both to test whether these additional measures are reliable predictors and to also evaluate their relative contribution overall to voice attractiveness.

## Methods

### Ethics Statement

Ethics approval for the collection of the stimuli was approved by the Institutional Review Board at the University of California, Berkeley. For the perception experiments, ethics approval was obtained from the Institutional Review Board at the University of California, Santa Cruz. Written informed consent was obtained from all participants.

### Stimuli

As part of a previous study [49], 30 male and 30 female voices were recorded reading the stimulus set shown in Table 1. Recordings were made at 44.1 kHz using a head-mounted microphone. Female talkers (mean age 24.2, range 18–57) and male talkers (mean age 24.1, range 18–47) did not differ significantly in age [*t*(51) = 0.05, *p* = ns]. The majority of the talkers were from California, and all talkers were from regions west of the Mississippi River. Select words containing the vowels /i a u/ were chosen for the current experiment because these sounds typically represent the maximum dispersion of the first and second formant frequencies of a talker's acoustic-phonetic vowel space. All tokens were normalized to have the same RMS amplitude and had silence trimmed by hand from the beginning and end of each file.

**Table 1:** Stimuli used in the experiment.

/u/	/i/	/a/
boot	deed	cot
dune	key	pod
hoop	peel	sock
toot	teal	sod
zoo	weave	tot

### Procedure

Each trial consisted of each of the 15 tokens from a single voice presented sequentially in random order with 500 ms between each sound file. Subjects listened to the voices over headphones at about 70 dB in a sound-attenuated booth. After the presentation of the fifteenth token subjects were asked to rate the attractiveness of the voice on a scale from 1–9, where 1 is unattractive and 9 is very attractive. Subjects were given no explicit instructions on how to judge “attractiveness” or rate the voices. Subjects could only respond after all tokens were presented and had an unlimited time to respond. The tokens from the next voice were presented 1000 ms after a response was logged. The order of voices was randomized for each subject and the experiment lasted approximately 35 minutes.

### Participants

Thirty native speakers of Californian English (15 females, 15 males) served as raters and received course credit or $10 for compensation. All reported normal hearing and had lived in California from toddlerhood.

### Analyses

#### Acoustic analysis

As noted above, the primary goal of the study was to expand the number of acoustic parameters used in studies of voice attractiveness. As in the previous studies, f0 and standardized formant position were measured for each talker. These values were averaged across all tokens for each talker and the standard deviation of f0 was also calculated from these measures. f0 and formant frequency measures were made via Praat 5.1.20 (Institute of Phonetic Sciences) using Gaussian windows with a 2.5 ms step size. Values were calculated separately for male and females where five formants within a 0–5 kHz range for the males and 0–5.5 kHz range for females. F1–F4 were used in calculating standardized formant position (following Puts et al. [14]; see also [50]). F5 was not reliably tracked and not included in any calculations. Following Bruckert et al. [38] the Harmonic-to-Noise ratio (HNR) was calculated in the 0–3.5 kHz range for each voice using the VoiceSauce package (http://www.ee.ucla.edu/~spapl/voicesauce/index.html). To these basic measures we added several additional acoustic measures, detailed below.

**Duration**: Males typically have shorter durations than females [46]. This was measured from the onset to offset of spectral energy for each word and averaged for each talker.

**Spectral Tilt**: This is a measure of voice quality [43] [51] [52] where, in general, higher values of tilt indicate breathier voices while lower values indicate creakiness. Several measures were taken using VoiceSauce: the short distance tilt measure of the amplitude of the first harmonic minus the amplitude of the second harmonic (H1–H2) and the longer distance measure of the first harmonic minus the peak amplitude of the first, second, and third formants (H1-A1, H1-A2, H1-A3, respectively).

**Jitter:** This is a local measure of deviation in periodicity; i.e. the averaged deviation of subsequent pitch periods, which makes this a measure of voice smoothness.

**Shimmer**: This is a local measure of variation in amplitude, i.e., the averaged deviation in amplitude of subsequent pitch periods, which makes this another type of measure which assesses voice smoothness.

#### Principal Component Analysis

Because many of these measures may be highly correlated and in order to reduce dimensionality, principal component analyses (PCA) were calculated for the vowel quality and voice quality measures. A full PCA with all acoustic measures was uninterpretable and impractical given the number of data points. Because male and female vocal tracts vary along a continuum of size, principal components for vowel quality were calculated on the entire dataset. The fact that PC1's proportion of variance was higher for the combined male and female vowel quality model as opposed to the separate PCA calculated on the female and male subsets was taken as an indication that the combined analysis offered a better account of the data. This PCA was unguided and used the F1–F3 Bark-transformed values for each vowel. Vowel PC1 accounted for just over 70% of the variance, with the remaining components accounting for considerably less variance, as is summarized in Table 2. Table 2 also provides the relative weightings and proportion of variance for each component, which are necessary to interpret what each component represents in terms of the acoustic measures. Vowel PC1 has positive loadings for all of the resonant frequencies, but is dominated by the F2 of /u/. Vowel PC4 has positive loadings for the F1 of /i/ and /u/ which suggests this component is largely representative of apparent vocal tract size, given the known relationship between the F1 of /i/ and /u/ and back cavity length [53].

**Table 2:** The cumulative proportion of variance accounted for and loadings from the PCA of vowel quality from F1/F2 measures.

	PC1	PC2	PC3	PC4	PC5	PC6
Standard Deviation	1.539	0.759	0.459	0.344	0.262	0.143
Proportion of variance	0.705	0.171	0.063	0.035	0.020	0.006
Cumulative variance accounted for	0.705	0.876	0.938	0.974	0.994	1.000
F1 /a/	0.340	−0.558	−0.181	−0.062	−0.724	−0.114
F1 /i/	0.222	−0.124	0.125	0.609	0.228	−0.704
F1 /u/	0.237	−0.095	0.042	0.678	0.007	0.688
F2 /a/	0.233	−0.528	−0.452	−0.229	0.634	0.094
F2 /i/	0.441	−0.166	0.806	−0.315	0.147	0.086
F2 /u/	0.728	0.599	−0.310	−0.113	−0.026	−0.038

Given fundamental differences in vocal fold vibration for males and females, separate PCAs were performed for male and female voice quality characteristics. These unguided PCA analyses included all of the voice quality and voice smoothness measures, i.e., H1–H2, H1-A3, HNR, jitter, and shimmer. The female analysis is summarized in Table 3. Female Voice PC1 represented 64.7% of the variance in the voice quality measures; Voice PC2 also accounted for a relatively large amount of the variance, nearly 25%. Voice PC3 through PC7 represented considerably less of the variance, and combined their contributions brought the model up to 100%. PC8 and PC9 provided very small contributions with shimmer and jitter weighted heavily for these higher components.

**Table 3:** The cumulative proportion of variance accounted for and loadings from the PCA of voice quality measures for female voices.

	PC1	PC2	PC3	PC4	PC5	PC6	PC7	PC8	PC9
Standard deviation	6.047	3.748	1.754	1.178	0.946	0.605	0.465	0.011	0.003
Proportion accounted for	0.6466	0.2484	0.0544	0.0245	0.0158	0.0065	0.0038	0.0000	0.0000
Cumulative proportion accounted for	0.6466	0.8950	0.9494	0.9739	0.9897	0.9962	1.0000	1.0000	1.0000
H1H2u	0.258	−0.033	−0.939	0.127	−0.118	0.144	−0.021	0.001	0.000
H1A1u	0.258	0.135	0.096	0.726	−0.142	−0.583	−0.139	−0.002	0.001
H1A2u	0.677	−0.012	0.296	−0.109	−0.569	0.343	0.025	−0.001	−0.001
H1A3u	0.618	−0.017	0.055	−0.072	0.762	−0.038	0.169	0.002	0.000
HNR35	0.052	0.925	−0.072	−0.306	−0.012	−0.112	−0.175	−0.005	0.001
CPP	−0.156	0.349	0.089	0.563	0.127	0.606	0.382	0.000	−0.002
Energy	0.012	−0.056	0.079	0.171	0.213	0.376	−0.880	−0.002	0.002
Jitter	0.000	−0.001	0.000	0.001	0.000	0.002	0.003	−0.174	0.985
Shimmer	0.000	−0.004	−0.001	0.000	0.002	0.000	0.003	−0.985	−0.174

From a general perspective, the male voice quality analysis is superficially similar to the females', and is summarized in Table 4. The male Voice PC1 accounted for 69% of the variance in the voice quality analysis, with the remaining components absorbing considerably less. Male voice PC7 was necessary to bring the entire model up to accounting for 100% of the cumulative variance, but like with the female voice models, while PC8 and PC9 accounted for only miniscule variance overall, they were strongly weighted with shimmer and jitter.

**Table 4:** The cumulative proportion of variance accounted for and loadings from the PCA of voice quality measures for male voices.

	PC1	PC2	PC3	PC4	PC5	PC6	PC7	PC8	PC9
Standard deviation	6.724	3.220	2.129	1.774	1.051	0.759	0.589	0.017	0.004
Proportion accounted for	0.693	0.159	0.069	0.048	0.017	0.009	0.005	0.000	0.000
Cumulative proportion accounted for	0.693	0.851	0.921	0.969	0.986	0.995	1.000	1.000	1.000
H1H2u	0.237	0.061	−0.177	0.719	−0.199	−0.292	−0.517	0.002	−0.003
H1A1u	0.418	0.184	−0.065	0.478	0.228	0.278	0.655	−0.001	0.002
H1A2u	0.648	−0.080	0.049	−0.319	0.578	−0.284	−0.234	0.002	−0.001
H1A3u	0.564	−0.142	0.271	−0.194	−0.633	0.377	−0.091	−0.002	0.001
HNR35	−0.018	−0.934	−0.007	0.170	−0.017	−0.203	0.241	0.002	0.000
CPP	−0.176	−0.111	0.722	0.293	0.354	0.379	−0.283	0.003	0.001
Energy	0.019	0.228	0.606	0.026	−0.220	−0.656	0.319	−0.001	−0.001
Jitter	0.000	0.001	0.000	−0.001	−0.001	0.003	0.003	0.442	−0.897
Shimmer	0.001	0.002	−0.001	−0.001	−0.003	0.000	0.001	0.897	0.442

## Results

### Listener ratings by gender

**Figure 1:**
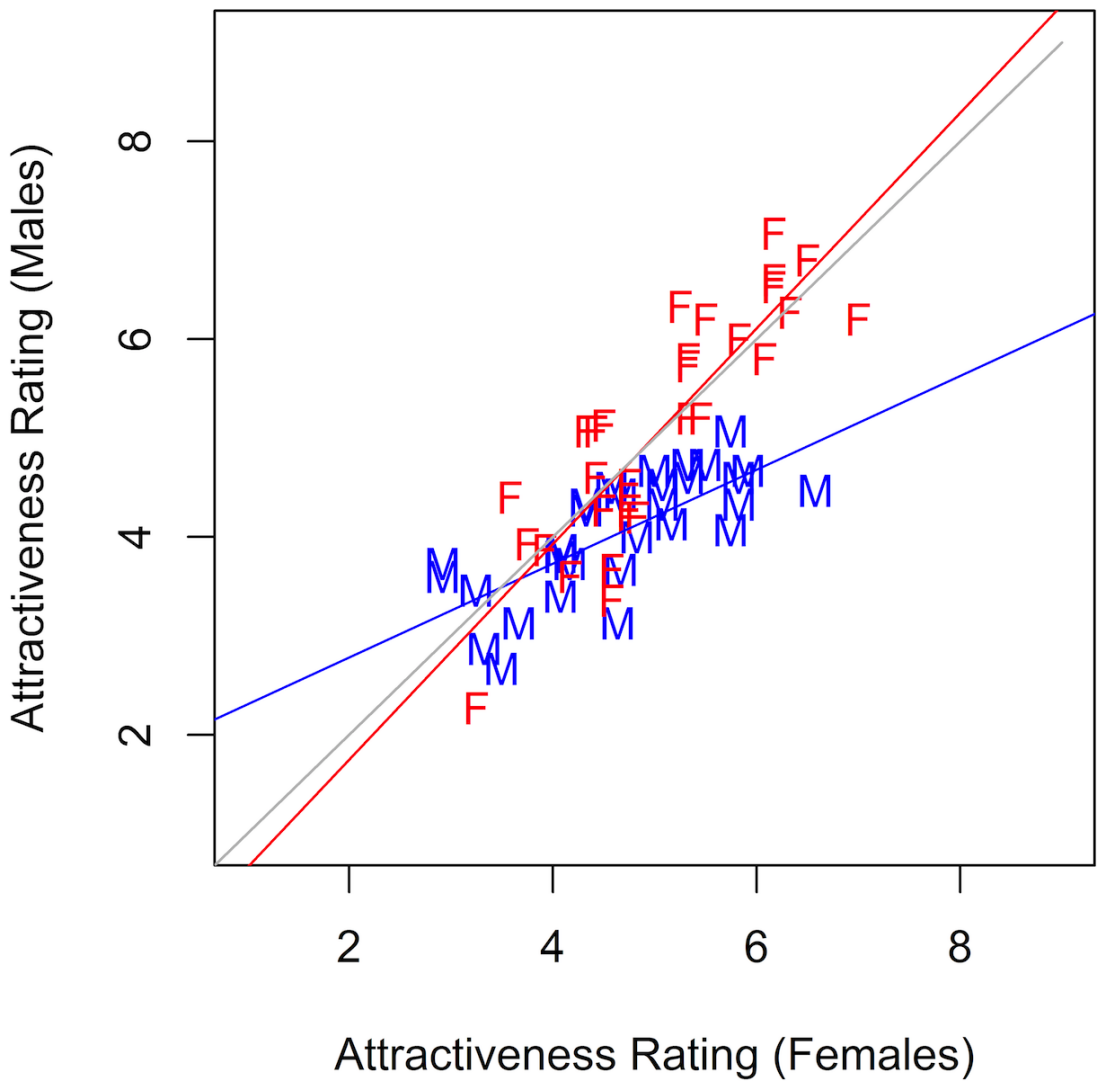
Correlations between Male and Female raters for Male (M, in blue) and Female (F, in red) voices.

Agreement between raters was assessed using Kendall's coefficient of concordance. The results are summarized in Table 5. Among all groups, for all listeners and voice genders there was strong inter-rater reliability; this was strongest for males rating females and weakest for males rating male voices. Table 6 summarizes the means and standard deviations of the ratings; ratings of male voices showed more variation than those for females and female voices were overall judged as more attractive. All listeners' judgments for each talker were averaged by listener gender and two Pearson product-moment correlation coefficients were computed to assess the relationship between male and female ratings of male voices and male and female ratings of female voices. Results show there was a strong correlation between both genders' ratings for both male voices (t[28] = 5.5, r = 0.74, p<0.001) and female voices (t[28] = 8.94, r = 0.86, p<0.001). This relationship is shown in Figure 1.

**Table 5:** Kendall's coefficient of concordance (*W*) for female and male voices from female and male raters.

Female Voices	*W*	*p*
Female raters	0.274	<0.001
Male raters	0.476	<0.001
Male Voices		
Female raters	0.274	<0.001
Male raters	0.185	<0.001

**Table 6:** Means and standard deviations for male and female voices and male and female raters.

Female Voices	*M*	*sd*
Female raters	5.05	1.89
Male raters	5.07	1.67
Male Voices		
Female raters	4.67	1.98
Male raters	4.05	1.99

The correlation shown in Figure 1, together with the information in Tables 4 and 5, indicate three main points. First, male and female raters agreed strongly on which voices are attractive and which are not for both genders. Second, this agreement tended to be stronger for the female voices; there is less agreement on the male voices which show both less inter-rater reliability and greater standard deviations. Finally, while males and females give female voices much the same attractiveness ratings, males rank fellow male voices as less attractive as a group than females do. These results suggest subtle differences in the ratings of male and female voices by the participants. While previous work has found that male listeners are essentially unwilling to rate other men with respect to attractiveness (providing uniformly low ratings for all voices, c.f. [32]), our male listeners generally agreed with female raters, although they provided slightly lower values. It is possible that males have less experience ranking male voices in terms of attractiveness or that they are unwilling to give male voices high attractiveness ratings.

### Predicting listener judgments

As this study is largely exploratory, stepwise linear regression models were used to predict listeners' attractiveness ratings. Before using the whole panoply of acoustic features, we examined the results of simple linear regression models for male and female voices using the more traditional measures of average pitch and formant position with the listeners' ratings as the dependent variable, with the hope of replicating previous findings. The model for the female voices is summarized in Table 7. Average f0 was not a significant predictor, but formant position was; listeners rated female voices with more dispersed formants, or apparently shorter vocal tracts, as more attractive. Overall, this model for the female voices was significant. The traditional model for the male voices is summarized in Table 8; contrary to the results for the female voices, it was not significant. The measures of average f0 and formant position did not significantly predict listeners' attractiveness ratings for the male voices.

**Table 7:** Predictors for the traditional regression model with female voices. F[2,27] = 5.07, Adjusted r^2^ = 0.22, p<0.05.

	Estimate	Std. Error	t value	p value
(Intercept)	6.73	1.98	3.4	<0.01
Average f0	−0.01	0.01	−1.34	0.19
Formant Position	3.27	1.03	−3.17	<0.01

**Table 8:** Predictors for the traditional regression model with male voices. F[2,27] = 1, Adjusted r^2^ = 0.02, p = 0.27.

	Estimate	Std. Error	t value	p value
(Intercept)	5.98	1.11	5.37	<0.001
Average f0	−0.01	0.01	−1.65	0.11
Formant Position	0.1	1.15	0.09	0.93

We computed a second round of linear regressions using the principal components described above as the independent variables. For each regression we used the appropriate principal components which brought the percentage of variance accounted for up to 95%, along with duration, f0 mean, and the standard deviation of f0. The formant position measure was highly correlated with the vowel quality PC1 [t(58) = 24.26, p<0.001, r = 0.95]. To avoid colinearity in the models, the formant position measure was not implemented in the reported analyses; we chose to use the vowel quality PC1 in lieu of the formant position measure because the principal component is a more comprehensive predictor based on a collection of several acoustic measures. We constructed models for female and male voices separately, using combined male and female principal components for the vowel quality and the separate components for voice quality. The variables for the final models were chosen using a backwards selection procedure with a criterion of p<0.15. Following this procedure, the two final models were then calculated with the remaining predictors. The first of these models, shown in Table 9, had listeners' attractiveness judgments of the female voices as its dependent variable and the other had the male voices; the male model is shown in Table 10. Zero-order correlations of the vocal attractiveness ratings and each acoustic variable are presented in Tables 11 for all voices and in Tables 12 and 13 for female and male voices, respectively, to aid in the interpretation of the results.

**Table 9:** Predictors for the regression model with female voices. F[4,25] = 9.4, Adjusted r^2^ = 0.54, p<0.001.

	Estimate	Std. Error	t value	p value
(Intercept)	7.93	1.57	5.05	<0.001
Average f0	−0.02	0.01	−2.21	<0.05
Vowel-PC1	0.48	0.17	2.85	<0.01
Vowel-PC3	0.52	0.28	1.82	0.08
Female Voice-PC3	−0.26	0.09	−2.95	<0.01

**Table 10:** Predictors for the regression model with male voices. F[7,22] = 7.23, Adjusted r^2^ = 0.60, p<0.001.

	Estimate	Std. Error	t value	p value
(Intercept)	5.67	0.63	8.96	<0.001
Vowel-PC1	−0.31	0.18	−1.75	0.09
Vowel-PC2	0.25	0.13	1.98	0.06
Vowel-PC3	−0.37	0.25	−1.52	0.14
Vowel-PC4	−1.75	0.33	−5.27	<0.001
Male Voice-PC1	0.03	0.02	1.85	0.08
Male Voice-PC4	−0.07	0.05	−1.46	0.16
Duration	−4.10	1.26	−3.26	<0.01

**Table 11:** Zero-order correlations between vocal attractiveness ratings and each acoustic measure for all 60 voices pooled together.

	df	t value	r	p value
f0	58	2.58	0.31	0.02
Duration	58	0.75	0.1	0.45
F2 /i/	58	4.84	0.54	<0.001
F2 /u/	58	4.42	0.51	<0.001
F2 /a/	58	0.92	0.12	0.36
F1 /i/	58	2.16	0.27	0.03
F1 /u/	58	1.79	0.23	0.08
F1 /a/	58	1.88	0.24	0.07
H1–H2	58	2.9	0.35	0.005
H1-A1	58	2.3	0.29	0.03
H1-A2	58	3.64	0.43	<0.001
H1-A3	58	3.06	0.37	<0.01
HNR	58	1.93	0.25	0.06
CPP	58	−2.94	−0.36	<0.01
Energy	58	−2.19	−0.28	0.03
Jitter	58	−0.9	−0.12	0.373
Shimmer	58	−0.32	−0.04	0.75

**Table 12:** Zero-order correlations between vocal attractiveness ratings and each acoustic measure for the 30 female voices.

	df	t value	r	p value
f0	28	−0.34	−0.06	0.74
Duration	28	1.28	0.24	0.21
F2 /i/	28	3.65	0.57	<0.01
F2 /u/	28	3.51	0.55	<0.01
F2 /a/	28	−0.25	−0.05	0.8
F1 /i/	28	1.44	0.26	0.16
F1 /u/	28	0.8	0.15	0.43
F1 /a/	28	−0.3	−0.06	0.76
H1–H2	28	1.2	0.22	0.24
H1-A1	28	1.99	0.35	0.06
H1-A2	28	2.91	0.48	<0.01
H1-A3	28	2.54	0.43	0.02
HNR	28	−0.22	−0.04	0.83
CPP	28	−2.46	−0.42	0.02
Energy	28	0.62	0.12	0.53
Jitter	28	−0.73	−0.14	0.47
Shimmer	28	−0.03	−0.007	0.97

**Table 13:** Zero-order correlations between vocal attractiveness ratings and each acoustic measure for the 30 male voices.

	df	t value	r	p value
f0	28	−1.46	−0.27	0.15
Duration	28	−0.94	−0.18	0.35
F2 /i/	28	0.48	0.09	0.63
F2 /u/	28	0.44	0.08	0.66
F2 /a/	28	−1.19	−0.22	0.24
F1 /i/	28	−4.45	−0.64	<0.001
F1 /u/	28	−3.33	−0.53	<0.01
F1 /a/	28	−0.63	−0.12	0.53
H1–H2	28	−0.07	−0.01	0.95
H1-A1	28	1.23	0.23	0.23
H1-A2	28	2.6	0.44	0.01
H1-A3	28	1.8	0.32	0.08
HNR	28	−0.95	−0.18	0.35
CPP	28	−2.25	−0.39	0.03
Energy	28	−0.41	−0.08	0.69
Jitter	28	1.13	0.21	0.27
Shimmer	28	1.68	0.3	0.1

While the female and male models share some features, they differ along several dimensions as well. The female model uses four predictors, but only three of these contribute significantly the model. The negative coefficient for average f0 for the female voices indicates that listeners rated female voices with lower fundamental frequencies as more attractive; the coefficient for this factor indicates, however, that the magnitude of this effect was miniscule. Vowel PC1 was a significant predictor for female voices. This component was positively loaded for all formant values, but was dominated by the F2 of /u/. The positive loadings for all formants may serve as an indicator of apparent-talker size with apparently smaller females being rated as more attractive, yet the weight of /u/ F2 for this component suggests that it is more strongly an indicator or dialect-specific vowel position with respect to the rest of the vowel space. More fronted productions of /u/ – that is, those that are higher in F2 – contribute to higher attractiveness ratings for female voices. Female Voice PC3 was also a significant contributor in predicting listeners' ratings. The negative coefficient of the voice quality principal component coupled with the negative and powerful loading of H1–H2 indicates that female voices exhibiting a breathier voice quality were judged as more attractive.

The male model included a much wider range of predictors than the female model, but only two of these contributed significantly: Vowel PC4 and average duration. Vowel PC4 is highly loaded with the F1 of /i/ and /u/, and the male model returns a negative coefficient for this factor. This indicates that listeners were more likely to rate a male voice as attractive if it had lower F1 values for /i/ and /u/, suggesting a larger back cavity. Male voices were also rated as more attractive if their productions were on average shorter in duration.

## Discussion

This experiment and analyses contribute major findings along two fronts. First, we show that male and female listeners largely agree with each other when rating vocal attractiveness. This is demonstrated by the strong correlations in ratings for male and female listeners and the significant inter-rater reliability. While this agreement is nearly one-to-one for female voices, males are reluctant to give fellow male voices high attractiveness ratings. There are several possible interpretations for this finding. It could simply be the case that males are not as experienced with rating male voices in this way. The low inter-rater reliability scores for the males rating male voices potentially support this. However, alternatively the ratings could be constrained by cultural norms relating to masculinity and perceived sexuality. These two accounts are not necessarily independent; cultural norms and taboos can limit the experience males have with rating the attractiveness of their fellow males. A final and related aspect of this is that the open-ended nature of the task and lack of specificity in instructing the subjects may have led the participants to approach the rating differently for different voices — i.e., as mate selection/sexual attraction versus likeability. A more directed task or more extensive post-task questioning of the participants could have resolved this ambiguity Overall, however, the general agreement we find amongst listeners illustrates that the perception of what constitutes attractive voices is shared between listeners of both genders.

Exactly which acoustic qualities are driving the shared attractiveness ratings is the second major finding. In using a wider variety of acoustic measures than previous studies, and then filtering out redundant colinearity with PCAs, we found that several parameters predict listener judgments. These parameters fit into measures that generally relate to apparent vocal tract size, apparent health or youthfulness, and typicality or membership in a speech community. We discuss our results with respect to each of these contributions in turn.

A major predictor for the attractiveness judgments for male voices illustrated the importance of lower formant frequencies, particularly the role of lower first formant frequencies for /i/ and /u/. This suggests that apparent vocal tract size matters for the perceived attractiveness of male voices. For most vowels, the first formant frequency is an indicator of back cavity length (i.e., the length of the vocal tract behind the articulatory constriction [53]), which is the portion of the vocal tract that differs most significantly across genders as a result of the lowering of the male larynx during puberty [54]. Lower first formant frequencies for /i/ and /u/ were judged as more attractive for males. It should also be noted that while F1 is generally a reliable indicator of back cavity length, this relationship is poorest with vowels like /a/ that have front and back cavities of near identical length [53]. Finally, we should note that previous studies on the effect of apparent vocal tract size have found mixed results. For example, Feinberg et al. [8] failed to find an independent effect for apparent vocal tract length, while studies such as Pisanski and Rendall [32] did find such an effect and Puts et al. [55] found a similar effect for dominance.

In this vein, while perceivable estimation of vocal tract size surfaced as a meaningful predictor in attractiveness judgments for male voices, formant position did not; however, that an estimation of back cavity length should be a better proxy for vocal tract length than formant spacing should not be surprising. A measure such as formant spacing is strongly affected by linguistic variance in vowel production, thus back cavity length as extracted from specific vowel productions is a more direct measure (and presumably more reliable). This may not be true for non-linguistic utterances (cries, sighs, screams, etc.) which may pattern more like the threat calls that produced effective vocal tract length proxies in Fitch's [56] primate research. Moreover, back cavity length is more directly related to the larynx lowering in post-pubescent males and thus a stronger cue to voice differences between males and females.

Our analysis does not necessarily contradict previous work which finds that simple apparent-size measures like formant position and f0 do play a role in perceived attractiveness. The results simply indicate that these factors do not significantly predict judgments of perceived attractiveness when additional acoustic measures are considered. To assess whether f0 and formant position play any role whatsoever in the attractiveness ratings for this voice corpus, we ran linear models with only f0 and formant position as potential predictors. These results returned a significant model for the female voices, but not the male voices. Moreover, while there is evidence for the universality of the cultural interpretation of f0 [26] [29], it is likely that different populations have different weights for its importance. Our results provide a concrete example of this: female voices with slightly lower average f0 values were rated as more attractive by listeners. Again, this finding does not directly contradict previous work which found slightly higher-than-average f0s were more attractive in female voices (e.g., [13] [20] [21] [22]). Rather, it seems that such findings may be less robustly generalizable across speaker and listener populations than previously assumed.

Female voices with breathier voice quality were rated as more attractive. This role of voice quality in the female model can be interpreted as either an indication of healthy or youthful larynges or as a generally feminine trait. Creaky voice qualities can often be associated with excessive smoking or drinking habits, in addition to more temporary ailments such as the common cold or laryngitis [57]. However, overall breathier voices are typical of female voices more generally (e.g. [42] [44]). Disentangling these two interpretations is not possible within this study. A further interpretation offered by Henton and Bladon [45] is that breathier voices might simulate arousal for females. We would suggest, however, that breathier voice qualities indicate younger and healthier larynges or femininity more generally, as opposed to a speech characteristic specifically associated with the indication of sexual arousal.

The results also point to the importance of local sociophonetic cues in assessing vocal attractiveness. For female voices, the largest contributor was a principal component associated with higher second formant frequencies for /u/. This pattern of /u/-fronting is characteristic of Californians, especially younger females [58] [59] [60] [61], and here it was found to be important to the attractiveness of the voice. We suggest that more attractive ratings for female voices with more fronted productions of /u/ is a preference for talkers who exhibit patterns similar to one's own speech; this is akin to the recurrent finding that perceivers have a preference for average faces [62]. Essentially, we can consider this to be a measure of speech conformity within a community.

For the male voices, averageness or typicality are also part of the duration result. Male voices with shorter durations were judged as more attractive. This result echoes what has been documented in the literature on male∼female differences; males typically have shorter durations than females [63] [64]. Judging male voices with shorter durations as attractive is again suggestive that attractiveness judgments are mediated by what is considered normal or average for a group.

Finally we should note that one major challenge is reconciling different results across experiments in the literature which synthetically manipulate the speech signal and those which retain a natural and therefore uncontrolled signal. By manipulating formant frequencies, researchers can, of course, clearly test hypotheses, but this can also lead researchers to synthesize combinations of acoustic-phonetic parameters that might not occur in natural speech, thus giving listeners tokens which do not approximate natural speech. Thus, both approaches are necessary for fully understanding the phenomena at hand.

## Conclusion

Our study expands on previous findings by demonstrating that acoustic-phonetic features relating to sexual dimorphism, apparent health and youthfulness, and community-based typicality collectively contribute to listeners' perception of vocal attractiveness. Moreover, we find that male and female ratings of attractiveness are highly correlated, which suggests that asking listeners to rate “how attractive a voice is” does not obligatorily involve an evaluation that conjures up associations with mate selection. Further research is needed to determine what kinds of characteristics, if any, are truly culturally universal. Crucially, the results of this study suggest that vocal attractiveness, like measures of attractiveness in other domains, is multi-dimensional in nature and involves the evaluation of multiple acoustically available and inferable traits.

These findings further reinforce that features of voices that indicate whether a talker is a typical male or female contribute to attractiveness ratings, whether derived or not from physiological differences. In the results described above, back cavity length appears to be a good predictor of male vocal attractiveness— a feature which is clearly derived from human sexual dimorphism. However, features such as duration are less clearly amenable to such an account. Moreover, the dominance of /u/-fronting in the prediction of attractiveness for female voices is similarly difficult to fold into a purely physiological account.

Given the correlational nature of this study and the relatively small sample sizes involved, the conclusions are necessarily tentative as we cannot causally link the acoustic variability in the voices to the listeners' ratings. The role of such multidimensional phonetic cues in judgments of vocal attractiveness need to be confirmed through experimental studies involving synthesis and other types of modification of the speech signal to independently vary the parameters we identify above. It is important that such work avoids essentializing a complex speech signal, which risks the creation of a misleading picture of how listeners perceive, categorize, and use the speech stream.
